# Berberine ameliorates blockade of autophagic flux in the liver by regulating cholesterol metabolism and inhibiting COX2-prostaglandin synthesis

**DOI:** 10.1038/s41419-018-0890-5

**Published:** 2018-08-01

**Authors:** Haidong Sun, Qian Liu, Hai Hu, Yisheng Jiang, Wentao Shao, Qihan Wang, Zhaoyan Jiang, Aihua Gu

**Affiliations:** 1 Center of Gallbladder Disease, Shanghai East Hospital, Institute of Gallstone Disease, Tongji University School of Medicine, Shanghai, China; 2 State Key Laboratory of Reproductive Medicine, Institute of Toxicology, Nanjing Medical University, Nanjing, China; 3 Key Laboratory of Modern Toxicology of Ministry of Education, School of Public Health, Nanjing Medical University, Nanjing, China; 4 Yucai High School, Shanghai, China

## Abstract

Excessive cholesterol contributes to the development of cardiovascular diseases. Berberine (BBR) has been reported to regulate cholesterol homeostasis. Here, we found that BBR could ameliorate the hepatic autophagic flux blockade caused by cholesterol overloading. The underlying mechanism included lowering hepatic cholesterol level, modulating the cholesterol distribution targeting the plasma membrane by decreasing sterol carrier protein 2 expression and inhibiting cyclooxygenase 2-mediated production of prostaglandin metabolites, which decreased the phosphorylation of Akt/mTOR. Our study provides evidences that BBR could be a therapeutic agent for protecting liver under cholesterol overloading via the regulation of autophagic flux.

## Introduction

Cholesterol overloading plays an important role in the development of metabolic disorders such as cardiovascular diseases^1,2^, which have been the leading cause of morbidity and mortality all over the world^3,4^. The liver is the key organ controlling cholesterol homeostasis in the body. Excessive cholesterol accumulation in hepatocytes may cause intensive lipotoxicity, such as endoplasmic reticulum (ER) stress^5^, lysosome impairment^6^, and mitochondrial function disruption^7^, and can further worsen metabolic disorders. Macroautophagy (referred to as autophagy) involves the formation of double-membrane autophagosomes that subsequently fuse with lysosomes for degradation^8^. Autophagy can participate in the metabolism of lipids, glucose, and proteins^9^, providing energy under the conditions lacking sufficient nutrients, such as starving^10^. Emerging evidence has revealed that autophagy plays a crucial role in lipid balance^11,12^ and that impaired autophagic flux can cause excessive lipid accumulation, severe hepatic oxidative stress, and inflammation. Notably, excessive lipid accumulation may induce dysfunction of autophagy and the subsequent lysomal degradation.

Berberine (BBR), an isoquinoline alkaloid isolated from *Berberis vulgaris* L., is commonly used to treat diarrhea. In recent years, its roles in cholesterol level reduction^13,14^, anti-inflammation^15^, and anti-ER stress^16^ in hepatocytes have been reported. To date, the known mechanisms by which BBR lowers cholesterol include the upregulation of hepatic low-density lipoprotein receptor^14^ and the inhibition of intestinal cholesterol absorption^17,18^. BBR has also been reported to play a role in the regulation of autophagy in adipocytes^19^. Whether BBR has a similar effect in hepatocytes has not been clarified.

Free cholesterol (FC) accumulation in hepatocytes was shown to block autophagic flux^20^. In the present study, we found that BBR could reverse the blockade of autophagic flux caused by high cholesterol in hepatocytes. BBR reduced accumulation of hepatocellular cholesterol, inhibited the expression of sterol carrier protein 2 (SCP2), thus decreased cholesterol distribution toward plasma membrane (PM) and down-regulated cyclooxygenase 2 (COX2)-mediated prostaglandin (PG) metabolism. All these effects taken together, inhibited the phosphorylation of Akt/mammalian target of rapamycin (mTOR) signaling, which is a master regulatory pathway of autophagy process^21^.

## Results

### BBR improved autophagic flux in the liver under cholesterol overloading

**Fig. 1:**
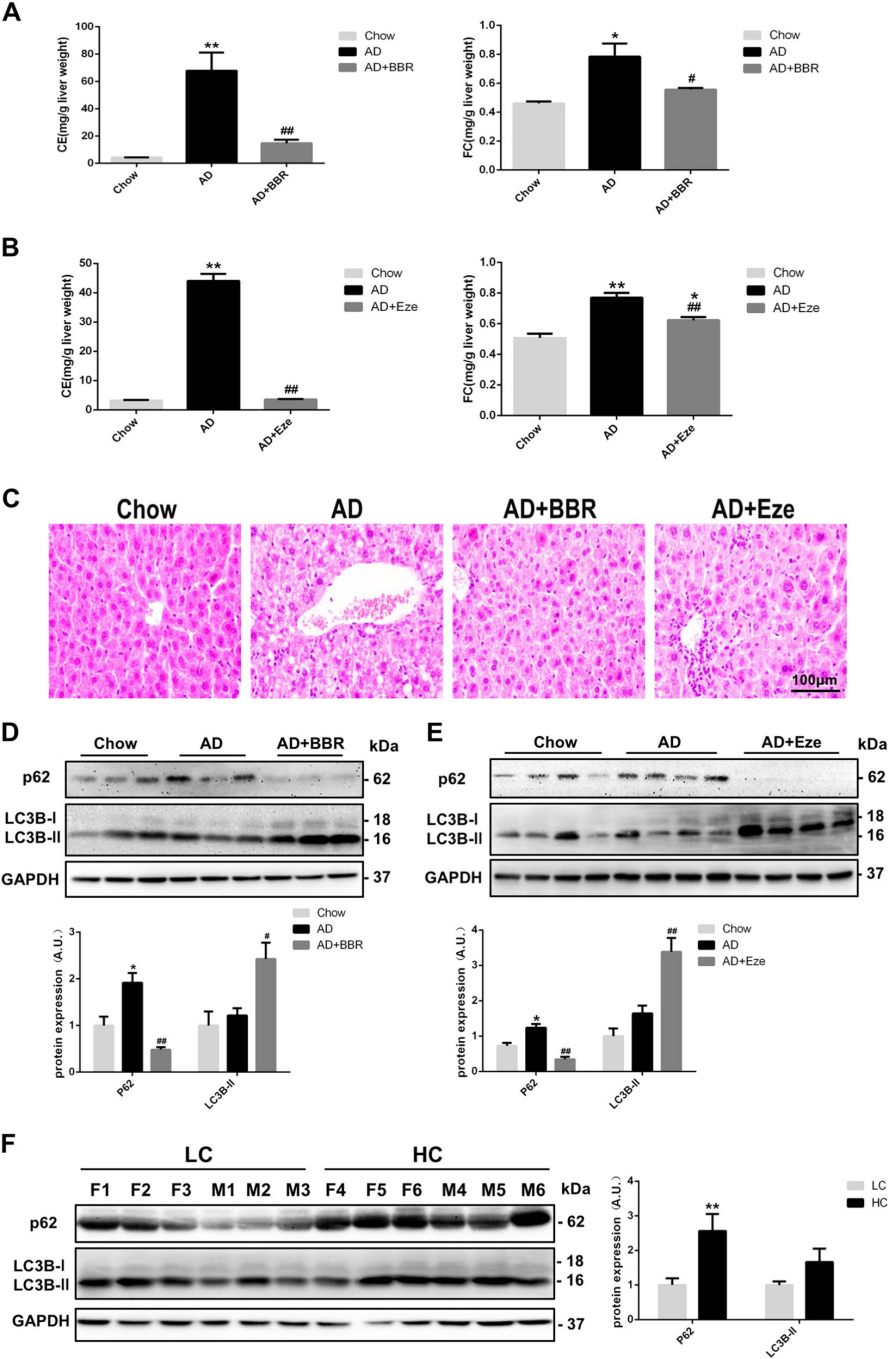
Effect of BBR on hepatic cholesterol accumulation and blockade of autophagic flux. **a**, **b** Cholesterol levels in the livers from mice fed with atherogenic diet (AD) or chow diet and treated with berberine (BBR) or ezetimibe (Eze). *p < 0.05 and **p < 0.01 when compared with chow diet; ^#^*p* < 0.05 and ^##^*p* < 0.01 when compared with AD. **c** H&E staining of liver tissues from each group (magnification: ×40). **d**, **e** LC3B-II and p62 protein levels in livers from mice fed with chow diet, AD, or treated with BBR or Eze. *p < 0.05 and **p < 0.01 when compared with chow diet; ^#^*p* < 0.05 and ^##^*p* < 0.01 when compared with AD. **f** Hepatic LC3B-II and p62 protein levels between patients with high cholesterol level (HC) and those with low cholesterol level (LC) in their livers. **p < 0.01 for the HC group compared with the LC group

Atherogenic diet (AD; Fig. 1a) and high cholesterol diet (HCD; Figure S1A) led to the accumulation of cholesterol in mouse liver. BBR treatment dramatically lowered both FC and cholesteryl ester in mice fed with these diets (Fig. 1a and Figure S1A). A similar effect was produced following treatment with ezetimibe (Fig. 1b), a pharmaceutical compound that lowers the cholesterol level by effectively inhibiting intestinal Niemann Pick C1 like 1 (NPC1L1)-mediated cholesterol absorption^22^. Hematoxylin and eosin (H&E) staining of mouse liver showed the appearance of hepatocyte ballooning due to the accumulation of lipid droplets that contained the bulk of CE and triglycerides (Fig. 1c). Both BBR and ezetimibe treatment improved the pathologic changes caused by AD.

We next examined the expression levels of two essential autophagy protein markers, microtubule-associated protein 1 light chain 3 (LC3) and SQSTM1 (p62). The accumulation of p62, indicating blockade of autophagic flux, was observed in the livers of mice fed with AD (Fig. 1d) as well as HCD (Figure S1B). Furthermore, in human liver biopsies (patient information listed in Supplementary Table 1), we also found blockade of autophagic flux in subjects who had high hepatic cholesterol content (Fig. 1f). In contrast, BBR treatment in mice significantly decreased the p62 protein level and increased the LC3B protein level (Fig. 1d). Similar findings were observed in mice treated with ezetimibe (Fig. 1e). These results suggested that BBR ameliorated the hepatic autophagic flux blockade related to cholesterol accumulation.

### BBR directly regulated autophagic flux in hepatic cells

**Fig. 2:**
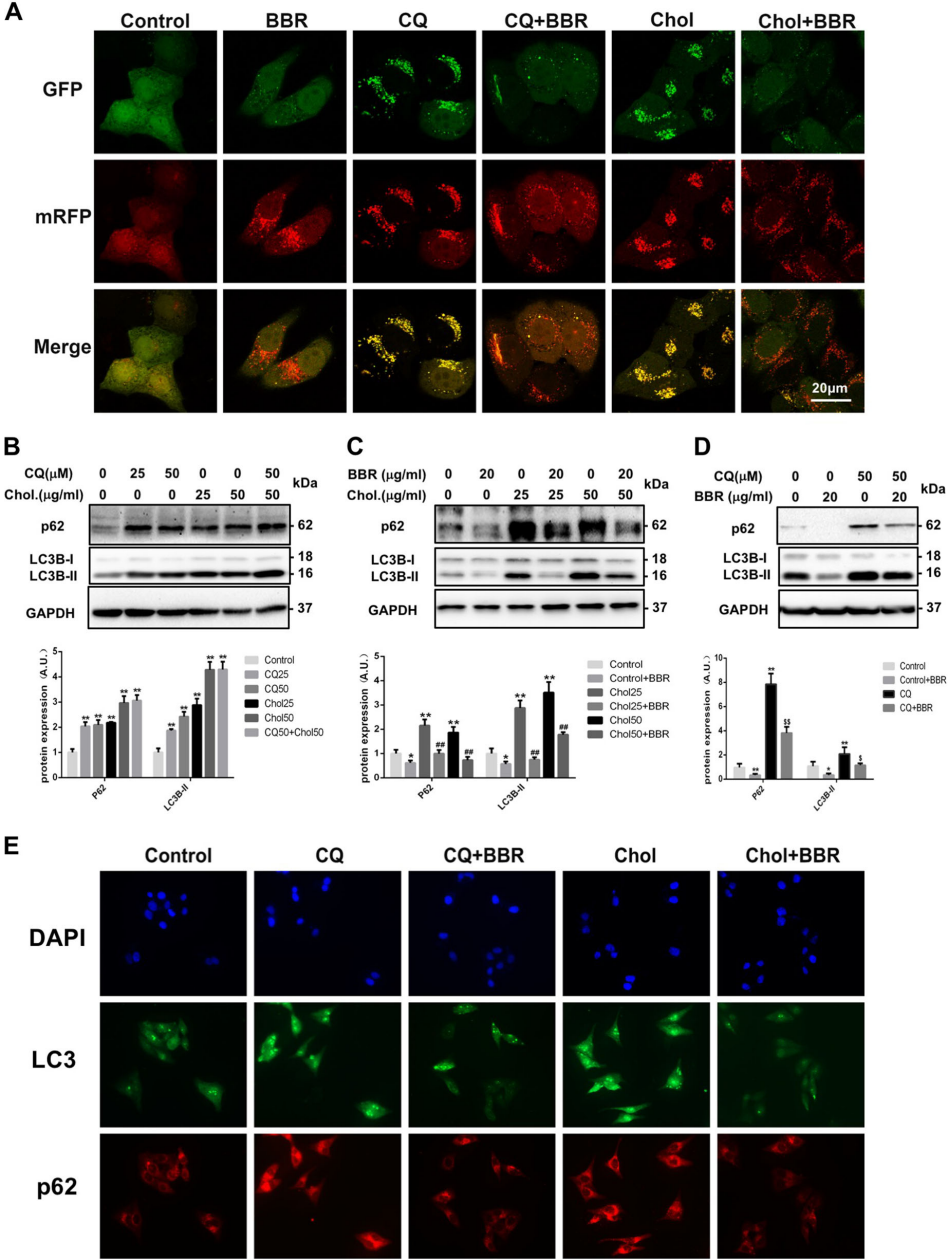
Effect of BBR treatment on cholesterol-induced blockade of autophagic flux in HepG2 cells. **a** Confocal microscopy examination of HepG2 cells expressing a tandem GFP-RFP-LC3 fusion protein treated with cholesterol (Chol, 50 µg/ml), chloroquine (CQ, 50 µg/ml), berberine (BBR, 20 µg/ml), or their combinations for 24 h. **b** LC3B-II and p62 protein levels in HepG2 cells treated with CQ, cholesterol (Chol), or a combination of CQ and cholesterol for 24 h. **c** LC3B-II and p62 protein levels in HepG2 cells treated with cholesterol (Chol), berberine (BBR), or a combination of cholesterol and BBR for 24 h. **d** LC3B-II and p62 protein levels in HepG2 cells treated with CQ, BBR, or their combination for 24 h. *p < 0.05; **p < 0.01: compared with control. ^##^*p* < 0.01: compared with cholesterol at the same concentration. ^$^*p* < 0.05 and ^$$^*p* < 0.01: compared with CQ. **e** Immunofluorescence staining of LC3B and p62 in HepG2 cells treated with cholesterol (50 µg/ml), CQ (50 µg/ml), BBR (20 µg/ml), or their combinations for 24 h

BBR treatment led to a significant increase in the appearance of green LC3B puncta in HepG2 cells (Figure S2A), suggesting the promotion of autophagosome/autolysosome formation. With electron transmission microscopy, we also observed typical structures of double-membrane formation, engulfment, and fusion with lysosomes and autolysosomes in the livers of mice treated with BBR (Figure S2B). The autophagic flux was further monitored in HepG2 cells expressing a tandem RFP-GFP-LC3B fusion protein. The green signal of the GFP quenches under acidic conditions, which occur after fusion with lysosomes; the red signal of the RFP serves as a marker for autolysosomes; and puncta with merged signals, shown as yellow, represent autophagosomes^23^. Cholesterol treatment increased the number of yellow puncta in the merged images, which is a sign of impaired autophagosome fusion with lysosomes (Fig. 2a). A similar phenomenon was observed when these RFP-GFP-LC3B fusion protein-expressing HepG2 cells were incubated with chloroquine (CQ), a lysosomal inhibitor used to block autophagic flux. In contrast, when these cells were incubated with BBR, the RFP proportions of the merged images significantly increased, suggesting BBR could facilitate the fusion of autophagosomes with lysosomes.

Increases in the expression levels of both the LC3B and p62 proteins were observed in HepG2 cells incubated with cholesterol (Fig. 2b) or with CQ. BBR, in contrast, decrease the LC3B and p62 proteins in HepG2 cells incubated with cholesterol (Fig. 2c) or CQ (Fig. 2d).

The results of immunostaining further showed a significant accumulation of both the LC3B and p62 proteins when hepatocytes were loaded with cholesterol (Fig. 2e). The same results were found after CQ treatment. BBR significantly decreased the expression of LC3B and p62 in these cells (Fig. 2e).

### BBR regulated the key proteins involved in cholesterol metabolism

**Fig. 3:**
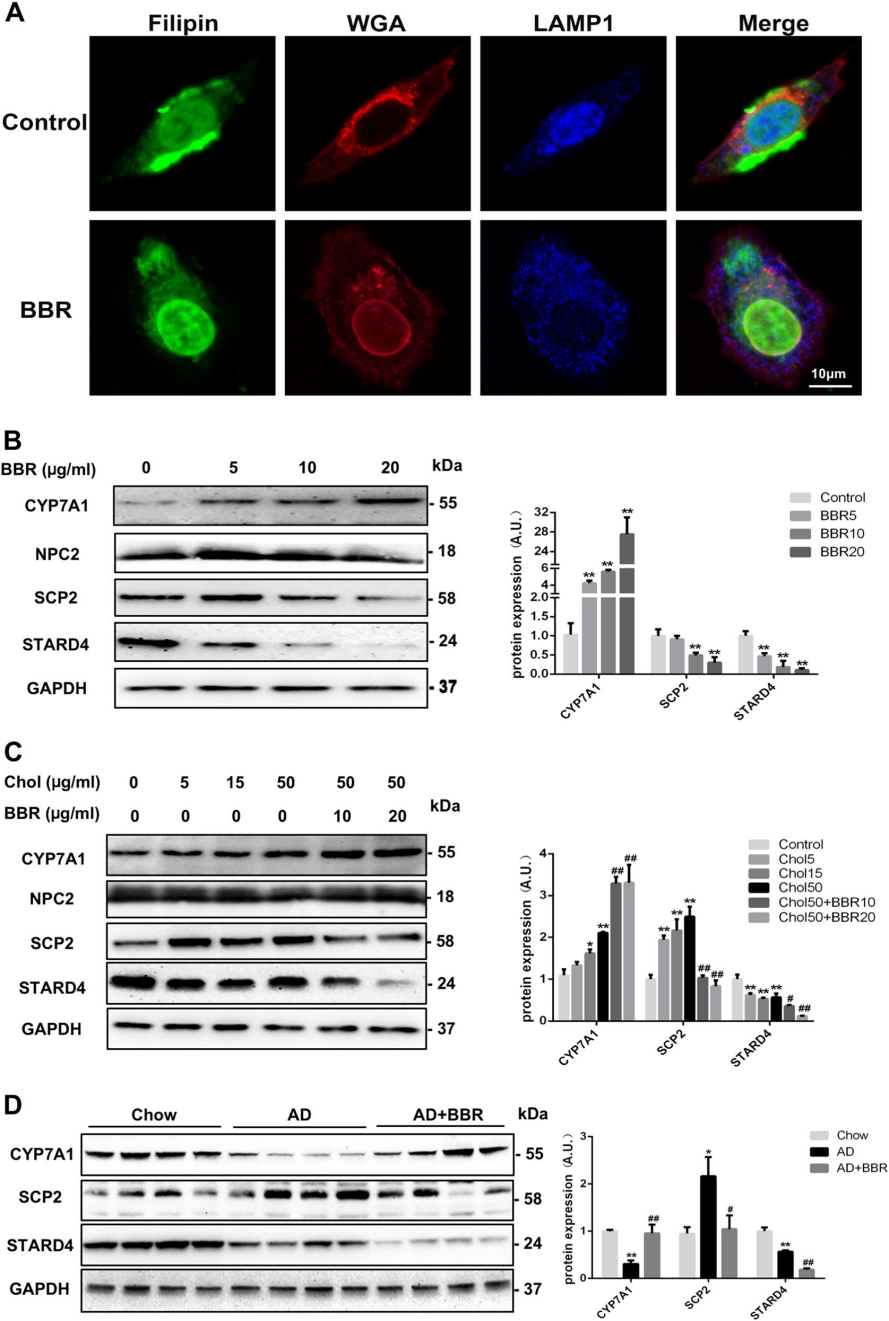
Cholesterol distribution and transporter protein expression in BBR-treated hepatocytes. **a** Confocal microscopy staining of the cholesterol distribution in HepG2 cells treated with berberine (BBR, 20 µg/ml for 24 h) as measured by filipin staining. Wheat germ agglutinin (WGA) and LAMP-1 were used for staining the membrane and lysosomes, respectively. **b** Expression of the CYP7A1, SCP2, STARD4, and NPC2 protein levels in HepG2 cells treated with different concentrations of berberine (BBR) for 24 h. **p < 0.01 compared with control. **c** Expression of the CYP7A1, SCP2, STARD4, and NPC2 protein levels in HepG2 cells treated with cholesterol alone (Chol) or cholesterol and BBR together for 24 h. *p < 0.05 and **p < 0.01 compared with control. ^#^*p* < 0.05 and ^##^*p* *<* 0.01 compared with cholesterol 50. **d** Expression of hepatic CYP7A1, SCP2, and STARD4 proteins in mice fed with different diets. *p < 0.05 and **p < 0.01 compared with chow diet. ^#^*p* < 0.05 and ^##^*p* < 0.01 compared with AD

The main reservoir for cholesterol in cells is the PM, which may contain 35%, and other intracellular organelles, such as the ER, may contain 5% of the total cholesterol^24^. Filipin, which selectively binds to FC^25^, is applied to visualize the distribution of cholesterol in hepatocytes. Using fluorescence microscopy, we found filipin signals were prominent in the PM under basal conditions, but intracellularly, they were weak and diffuse (Fig. 3a). BBR treatment dramatically decreased the filipin signals in the PM and increased its co-localization with LAMP-1, a marker for late endosomes and lysosomes (Fig. 3a). These data suggested that BBR triggered the re-distribution of intracellular FC.

Several transporter proteins are involved in intracellular cholesterol trafficking^26^. SCP2, the mature form of SCPX, is involved in the transport of cholesterol from the ER to the PM^27^. StAR-related lipid transfer domain containing 4 (STARD4) is involved in the efflux of cholesterol from late endosomes^28^. Both proteins were down-regulated in HepG2 cells treated with BBR (Fig. 3b), also in cells treated with cholesterol (Fig. 3c). However, no difference in the expression of Niemann Pick C2 (NPC2), a protein involved in cholesterol trafficking between late endosomes and lysosomes^29^, was observed between cells treated with BBR and controls. The downregulation of hepatic SCP2 and STARD4 by BBR was further confirmed in mice fed with AD (Fig. 3d).

The conversion of cholesterol into bile acids is a crucial way to remove excessive cholesterol from the body. Cholesterol 7α-hydroxylase (CYP7A1) is the rate-limiting enzyme for this process. Interestingly, we found that BBR dose-dependently upregulated the expression of CYP7A1 in HepG2 cells (Fig. 3b), in the presence or absence of cholesterol (Fig. 3c), and in mouse livers (Fig. 3d). Collectively, these data showed that BBR modulated cellular cholesterol by alleviating intracellular cholesterol trafficking targeting PM and by promoting the degradation of cholesterol into bile acids.

### BBR activated autophagy via inhibition of AKT/mTOR phosphorylation

**Fig. 4:**
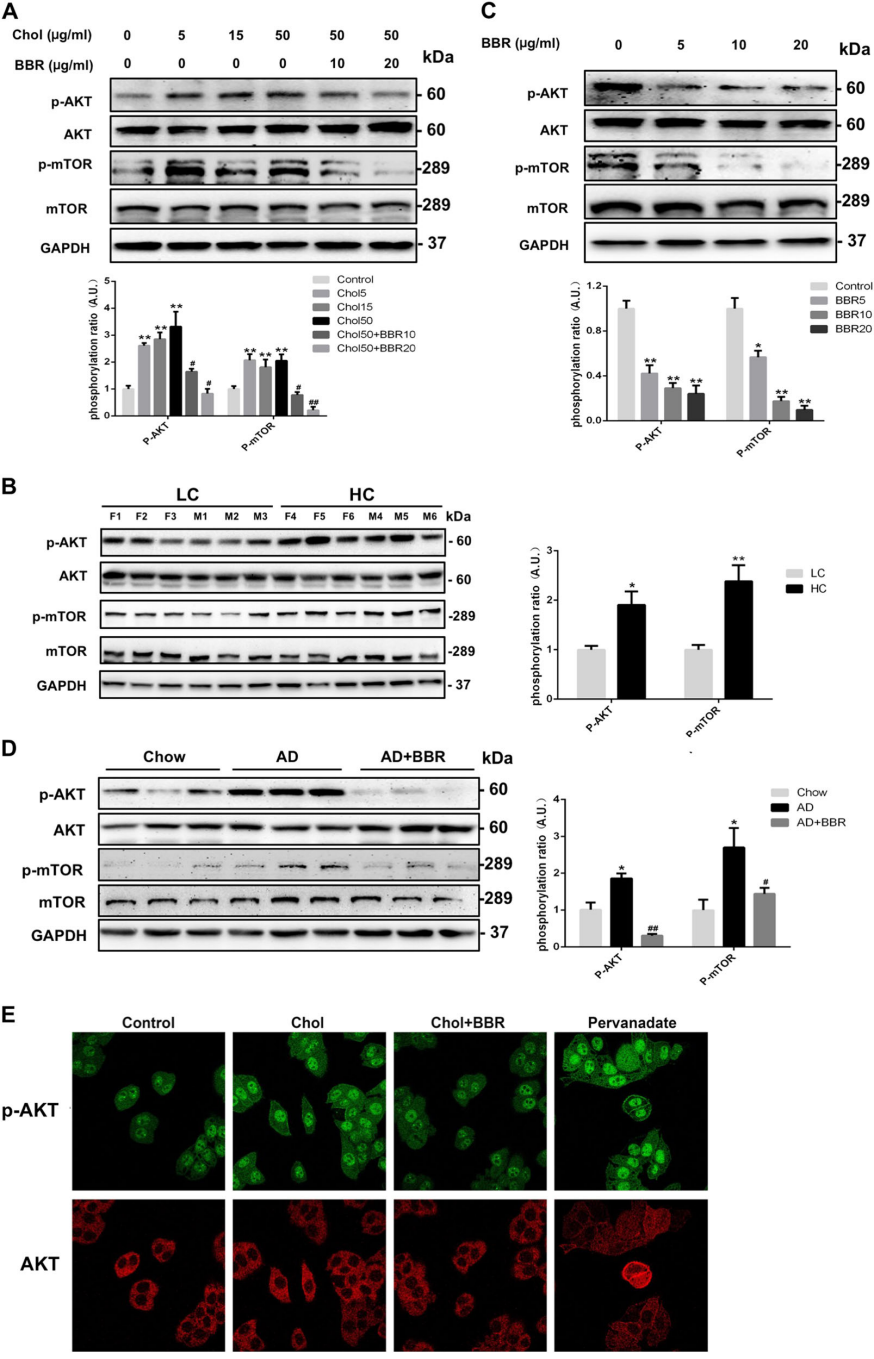
Effect of BBR on AKT and mTOR phosphorylation. **a** Expression of phosphorylated AKT and mTOR in HepG2 cells treated with cholesterol (Chol) or cholesterol + berberine (BBR) for 24 h. **p < 0.01 compared with control. ^#^*p* < 0.05 and ^##^*p* < 0.01 compared with cholesterol 50. **b** Hepatic expression of phosphorylated AKT and mTOR protein levels between patients with high cholesterol level (HC) and those with low cholesterol level (LC) in their livers. *p < 0.05 and **p < 0.01 for the HC group compared with the LC group. **c** Expression of phosphorylated AKT and mTOR in HepG2 cells treated with different concentrations of BBR for 24 h. *p < 0.05 and **p *<* 0.01 compared with control. **d** Expression of phosphorylated AKT and mTOR in the livers of mice fed with different diets. *p *<* 0.05 compared with chow diet. ^#^*p* < 0.05 and ^##^*p* < 0.01 compared with AD. **e** Confocal microscopy images of p-AKT activation in HepG2 cells treated with cholesterol (Chol: 50 µg/ml) alone or with cholesterol (Chol: 50 µg/ml) + BBR (20 µg/ml) for 8 h; pervanadate (50 µM), a strong AKT dephosphorization inhibitor, was used as a positive control

AKT/mTOR is an important pathway regulating the autophagy process^21,30^. Our data showed that cholesterol overloading led to an activation of AKT and mTOR phosphorylation in HepG2 cells (Fig. 4a) as well as in human livers (Fig. 4d). Treatment with BBR inhibited the phosphorylation of both AKT and mTOR (Fig. 4b) and reversed the cholesterol-induced activation of AKT/mTOR in both HepG2 cells (Fig. 4a) and mouse livers (Fig. 4c and Figure S1B). No alteration of ERK or AMPK phosphorylation by BBR was observed in the present study (data not shown). Our data indicated that BBR could induce autophagy through the inhibition of AKT/mTOR phosphorylation.

AKT activation mainly takes place at the PM^31^, particularly at lipid raft sites where cholesterol is rich^32^. Using pervanadate, a potent AKT activator^33^, we observed localization and activation of AKT at the PM (Fig. 4e). Cholesterol loading also activated AKT phosphorylation at the PM, suggesting the recruitment of AKT to the PM. In contrast, BBR treatment attenuated the phosphorylation of AKT at the PM (Fig. 4e).

### BBR modulated autophagic flux through the inhibition of PGE2 synthesis

**Fig. 5:**
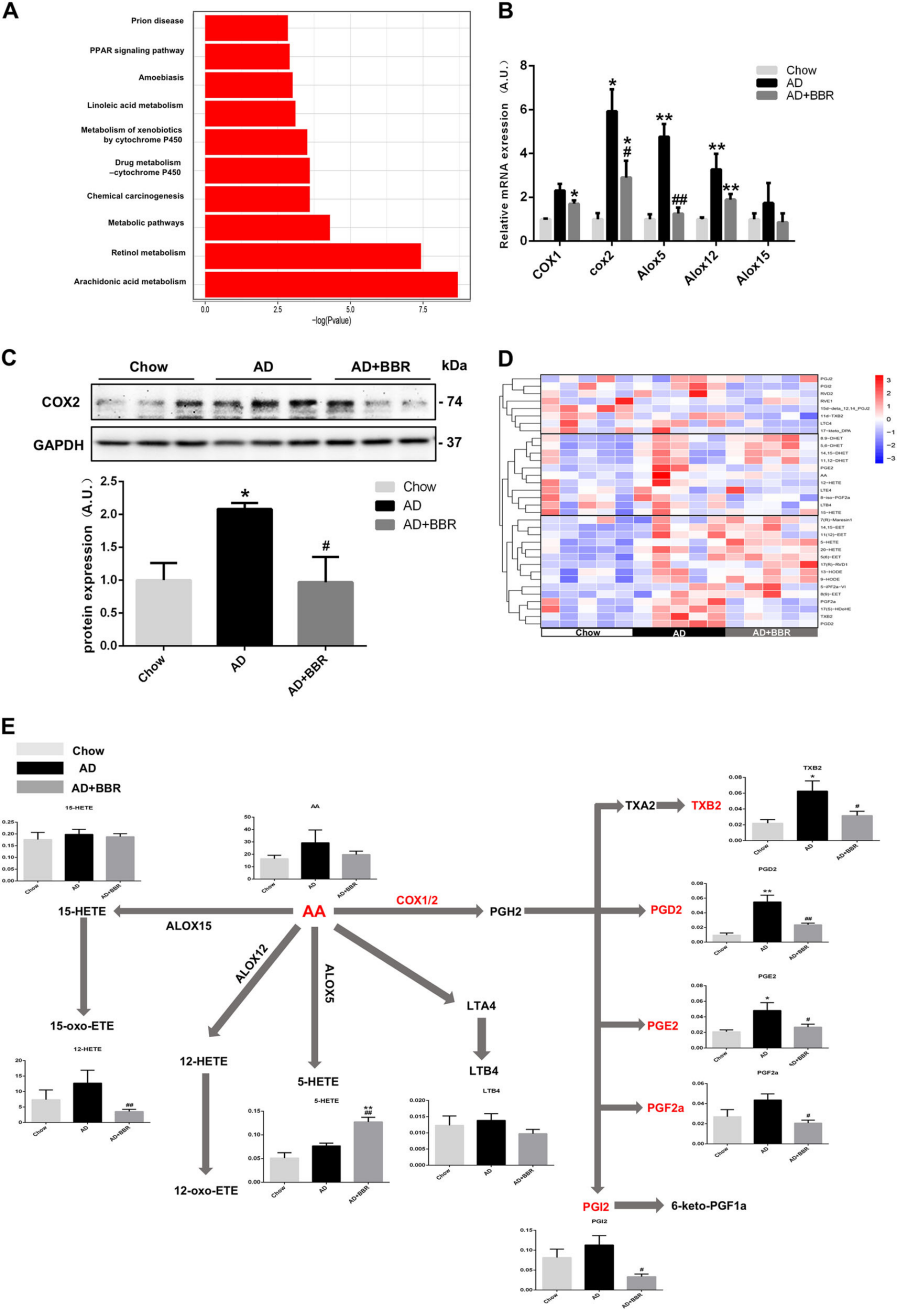
Regulation of hepatic COX2/prostaglandin metabolism by BBR. **a** KEGG analysis of the top 10 pathways in the livers of mice fed with atherogenic diet (AD) alone or with berberine (AD + BBR). **b** Expression of key genes involved in the hepatic metabolism of arachidonic acid and prostaglandin metabolites. **c** Changes of the COX2 protein levels in the livers of mice fed with different diets. *p < 0.05 compared with chow diet. ^#^*p* < 0.05 compared with AD. **d** Heatmap show of arachidonic acid and prostaglandin metabolites in the livers of mice fed with chow, AD, and AD + BBR. **e** Levels of arachidonic acid and prostaglandin metabolites among groups (unit: ng/mg liver). *p < 0.05 and **p < 0.01 compared with chow diet. ^#^*p* < 0.05 and ^##^*p* < 0.01 compared with AD

A KEGG analysis of the RNA-seq data was performed to further unravel the potential mechanisms of BBR. Arachidonic acid (AA) metabolism was the pathway most regulated by BBR in mice fed with AD (Fig. 5a). The hepatic mRNA expressions of key genes involved in AA metabolism were increased by AD consumption and were down-regulated by BBR (Fig. 5b). Additionally, both the mRNA and protein levels of COX2 were increased by AD and lowered by BBR treatment (Fig. 5b, c). Target metabolomics analysis of the AA and PG metabolites revealed similar changes as COX2 expression in mice fed with AD and in those treated with BBR (Fig. 5d, e).

**Fig. 6:**
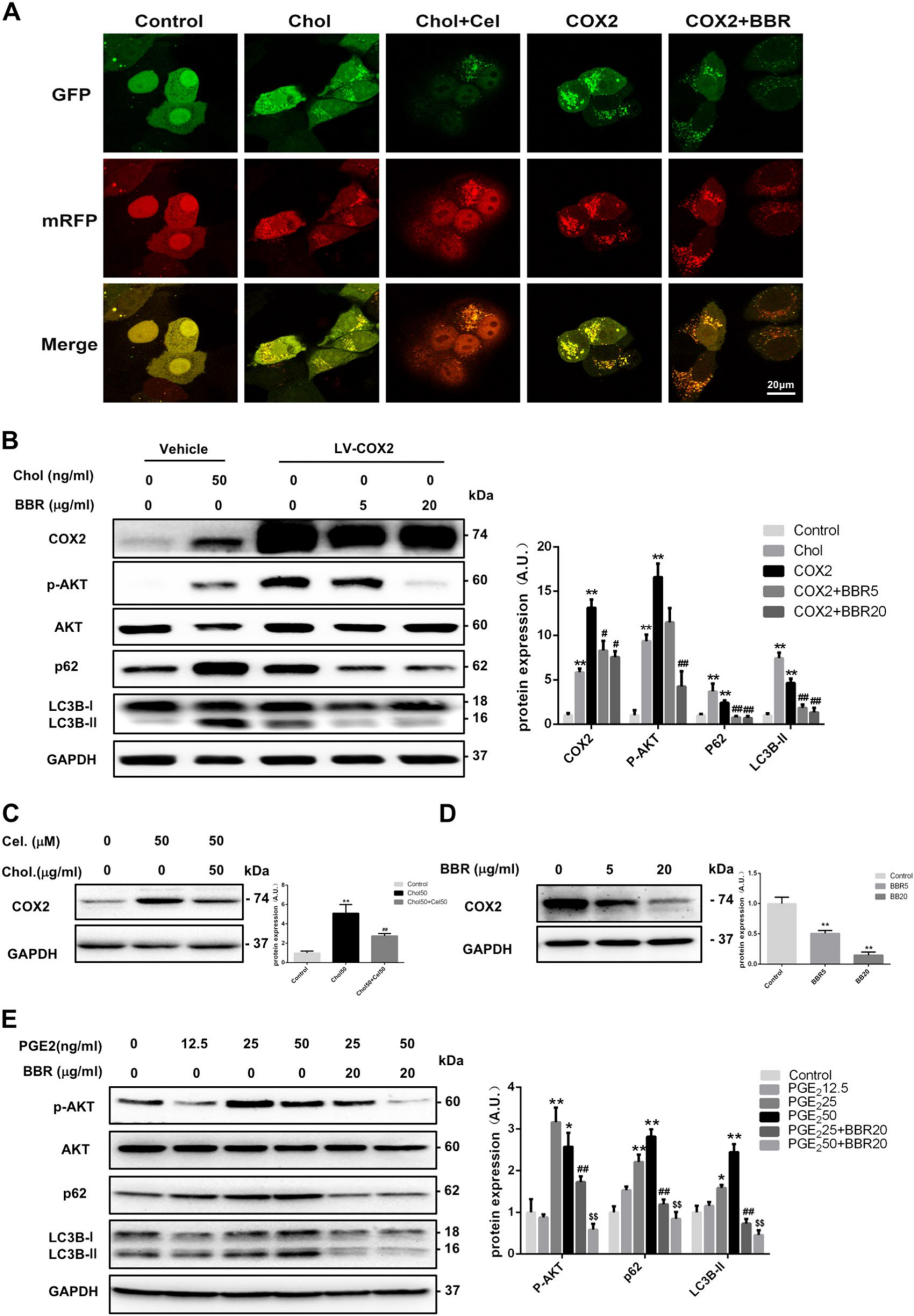
COX2 expression and autophagic flux in BBR-treated HepG2 cells. **a** Confocal microscopy examination of HepG2 cells expressing a tandem GFP-RFP-LC3 fusion protein that were treated with cholesterol (Chol, 50 µg/ml) with/without celecoxib (Cel; 50 µM), or subjected to lentivirus-mediated overexpression of COX2 protein with/without berberine (BBR, 20 µg/ml) treatment for 24 h. **b** Protein levels of COX2, p-AKT, LC3II, and p62 in HepG2 cells treated with cholesterol or lentivirus-mediated overexpression of COX2 with BBR for 24 h. **p < 0.01 compared with control. ^#^*p* < 0.05 and ^##^*p* < 0.01 compared with LV-COX2. **c**, **d** Expression of COX2 protein in HepG2 cells treated with cholesterol, cholesterol + Cel, or BBR for 24 h. **p < 0.01 compared with control. ^##^*p* *<* 0.01 compared with cholesterol. **e** Expression of p-AKT, p62, and LC3II proteins in HepG2 cells treated with different concentrations of PGE2 alone or together with BBR for 24 h. *p < 0.05 and **p < 0.01 compared with control. ^##^*p* < 0.01 compared with PGE_2_ 25. ^$$^*p* < 0.01 compared with PGE_2_ 50

In cholesterol-overloaded HepG2 cells, the blockade of autophagic flux (Fig. 6a) was accompanied by an induction of COX2 expression (Fig. 6b). Treatment with celecoxib, a selective COX2 inhibitor, lowered the COX2 expression (Fig. 6c) and ameliorated the cholesterol-induced blockade of autophagic flux (Fig. 6a). Overexpression of COX2 in HepG2 cells led to significantly increased phosphorylation levels of AKT and mTOR (Fig. 6b), resulting in the blockade of autophagic flux (Fig. 6a). Treatment with BBR partly ameliorated this blockade (Fig. 6a) by inhibiting the expression of COX2 protein (Fig. 6b) and the phosphorylation of AKT/mTOR as well (Fig. 6d and Figure S3).

PGE2 comprises the majority of metabolites in PG metabolism and plays a vital role in hepatic inflammation in response to cholesterol accumulation. Interestingly, the autophagic flux in HepG2 cells was dose-dependently blocked by PGE2, as suggested by the accumulation of both the LC3B and p62 proteins. In contrast, BBR treatment in PGE2-treated cells restored the autophagic flux (Fig. 6e). We also observed that PGE2 activated AKT phosphorylation and that BBR inhibited this process (Fig. 6e). These data suggested that PG metabolites might be the mediators that inhibited the autophagy process in hepatocytes, which was efficiently assuaged by BBR treatment.

## Discussion

**Fig. 7:**
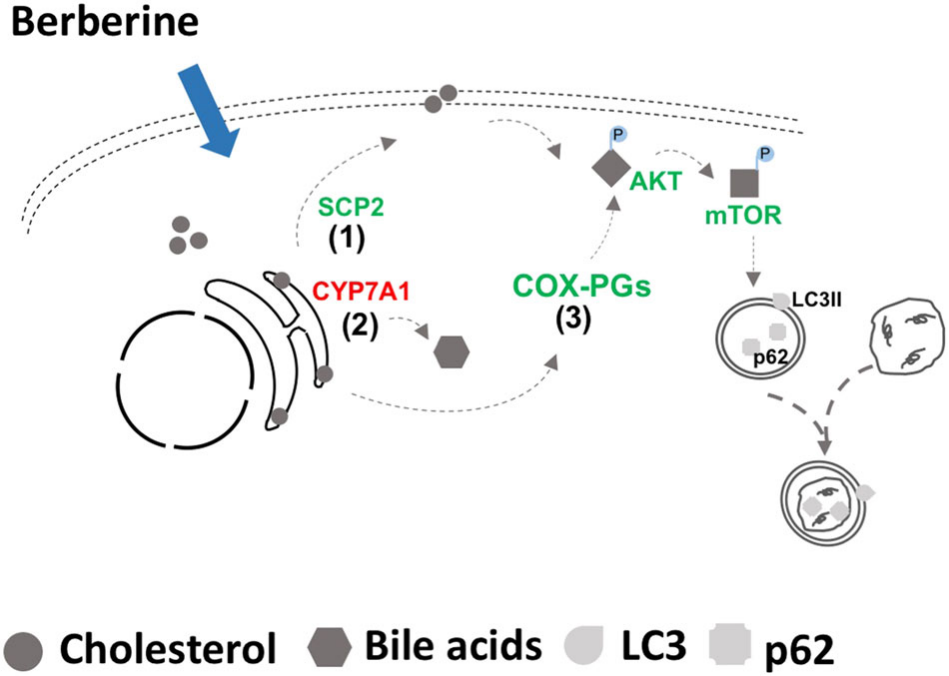
Schematic diagram of the mechanisms by which BBR ameliorates the blockade of autophagic flux induced by cholesterol overloading. BBR could (1) down-regulate SCP2 and inhibit cholesterol trafficking toward plasma membrane; (2) activate CYP7A1 expression and induce bile acid synthesis; and (3) mitigate COX2-mediated prostaglandin synthesis, which together led to the inhibition of AKT/mTOR phosphorylation and improved autophagic flux

Hepatic cholesterol overloading blocks autophagic flux via the activation of AKT/mTOR signaling. Here, we found that BBR treatment down-regulated the phosphorylation of AKT/mTOR and ameliorated the blockade of hepatic autophagic flux (Fig. 7). This effect was connected with the ability of BBR to reduce cellular cholesterol loading, to inhibit cholesterol trafficking toward the PM, and to mitigate COX2-mediated PG synthesis, which together led to the inhibition of AKT/mTOR phosphorylation.

The cellular cholesterol level is important for autophagy regulation^20,34^. In hepatic cells, we found that FC accumulation impaired autolysosome function and clearance, as evidenced by increase of p62 proteins and the accumulation of merged yellow puncta in cells expressing the RFP-GFP-LC3 fusion protein. This finding is in agreement with previous observations by Liu et al.^34^ and Wang et al.^20^. However, the response to cholesterol appears to be cell type-specific. In macrophages, unlike in hepatocytes, cholesterol accumulation induced autophagy to attenuate cholesterol toxicity by increasing the cholesterol efflux^35^. One important finding of our study is that BBR is capable of reversing the blockade of autophagic flux caused by cholesterol overloading in hepatocytes with decreased p62 protein levels. This effect was consistently observed both in vitro and in vivo.

The AKT/mTOR axis is a master signaling pathway in autophagy regulation^30^. We found that cholesterol overloading promoted AKT phosphorylation at the PM, which is a common mechanism of AKT signaling^36^. BBR treatment attenuated the translocation of AKT toward the PM as well as its phosphorylation. Our data provided evidences that BBR regulated the AKT/mTOR phosphorylation through three ways. First, BBR reduces the overall exogenous hepatic cholesterol level through inhibiting intestinal cholesterol absorption^17,18^. BBR was previously shown to inhibit the esterification of FC by ACAT2 in intestinal epithelium cells and to interfere with luminal cholesterol micelle formation^17^. AD consumption caused six-fold increase of CE in mouse livers, whereas BBR treatment reduced the hepatic CE level to about two-fold higher compared with that in mice fed with a regular chow diet. As a result, the blockade of autophagic flux caused by HCD was greatly ameliorated by BBR treatment. Ezetimibe is an even more potent inhibitor of intestinal cholesterol absorption^22^, and it almost normalized the hepatic cholesterol content to a level similar to that in mice fed with chow diet. Additionally, ezetimibe treatment produced a similar amelioration of the cholesterol-induced autophagic flux blockade in mouse livers.

Second, BBR plays a direct role in regulating cholesterol trafficking in hepatocytes. Upon cholesterol overloading, the FC rapidly distributed to the PM, which is a reservoir for cholesterol and is where AKT phosphorylation activated^25^. Notably, depletion of FC at the PM would decrease the phosphorylation of AKT and mTOR^31,37^. BBR treatment decreased both the expression of SCP2 in hepatocytes and the PM cholesterol level, as evidenced by the filipin staining. Autophagy activation by itraconazole was reported to occur through inhibiting SCP2 expression, reducing cholesterol in the PM, and inhibiting phosphorylation of AKT/mTOR^34^. Although BBR treatment decreased cholesterol transportation to the PM, cholesterol did not accumulate in organelles such as the ER, endosomes, or lysosomes, partly due to a simultaneous activation of CYP7A1, which is responsible for the conversion of cholesterol into bile acids. The capability of BBR to induce CYP7A1 was also found in both a human hepatoma cell line^38^ and rodents^39,40^. Moreover, the induction of CYP7A1 expression by genetic modulation or cholestyramine treatment resulted in the activation of autophagic flux in hepatocytes^20^.

Third, BBR affects the phosphorylation of AKT through the regulation of PG metabolism. PGH2 is converted from AA by cyclooxygenase and then further metabolized into D, E, F, and I series PGs by PG-synthases and isomerases. As the rate-limiting enzyme in PG synthesis, COX2 is induced by cytokines, growth factors, and tumors^41^. The AD consumption is known to induce hepatic inflammation via the upregulation of pro-inflammatory cytokines, such as tumor necrosis factor-α (TNFα), interleukin (IL)1β, and IL6. As a result, COX2-mediated PG metabolites are elevated in the liver of mice fed with AD. We, for the first time, showed that BBR treatment inhibited hepatic COX2 expression and reduced PG metabolite accumulation. BBR treatment has been previously reported to decrease obesity-induced liver inflammation^42^ and to reduce ER stress during liver steatosis^16^. Increase of PGE2^43,44^ as well as TNFα^45^ and IL1β^46^ was shown to induce COX2 expression facilitating the activation of AKT. Here, we observed that PGE2 dose-dependently activated AKT phosphorylation and blocked the autophagic flux in HepG2 cells, which was reversed by BBR treatment.

Collectively, the present study revealed that BBR could improve autophagic flux in hepatocytes by decreasing AKT/mTOR phosphorylation through the mechanisms depicted in Fig. 7. The results of our study provided a novel mechanism for how BBR protects liver through the activation of autophagy process, in addition to its role in regulating the cholesterol level. BBR might be a therapeutic option for treating conditions in which the liver is burdened by cholesterol loading.

## Materials and Methods

### Regents and materials

Berberine chloride (BBR), CQ, wheat germ agglutinin (WGA), water-soluble cholesterol (in methyl-beta-cyclodextrin), prostaglandin E (PGE2), and dimethyl sulfoxide (DMSO) were purchased from Sigma-Aldrich (Supplementary Table 2). All information concerning the antibodies used in this study are listed Supplementary Table 3.

### Animal studies

Mouse food for AD (containing 1.25% cholesterol and 0.5% cholic acid) and HCD (containing 1.25% cholesterol) were purchased from Trophic Animal Feed High-Tech Co. Ltd (Nantong, China). Adult male C57BL/6 mice (8-week-old, Shanghai SLAC Laboratory Animal Co., Ltd, Shanghai, China) were randomly allocated to groups fed with different diets (*n* = 5 mice/group): chow diet, AD, HCD, AD + BBR (100 mg/kg/day, by gavage), AD + ezetimibe (5 mg/kg/day, by gavage), or HCD + BBR (100 mg/kg/day, by gavage) for 8 weeks. Upon sacrifice, blood samples and liver tissues were collected. All the experiment protocols were approved by the Animal Use and Care Committee of Shanghai East Hospital, Tongji University School of Medicine.

### Cell culture

HepG2 cells were cultured in Dulbecco's modified Eagle's medium containing 10% fetal bovine serum. Working solution of pervanadate was prepared using a previously described method^47^. Before any treatment, equal numbers of cells were plated in six-well plates and cultured until all wells reached ~70% confluence. These cells were then washed with cold phosphate-buffered saline (PBS) at least twice and subjected to BBR, CQ, cholesterol, PGE2, or pervanadate treatment. Finally, the cells were either harvested for western blot analysis or fixed for immunofluorescence staining.

### mRFP-GFP-LC3 adenovirus transduction

The mRFP-GFP-LC3 adenovirus was obtained from HanBio Inc. (Shanghai, China). HepG2 cells were infected with adenoviral particles at a multiplicity of infection (MOI) of 10 for 24 h. After complete adenovirus transduction, the cells were washed with PBS and subjected to CQ, cholesterol, or BBR treatment. Following the treatment, 4% paraformaldehyde was used to fix the cells, and a Zeiss LSM-710 confocal microscope was used to observe the number of GFP-positive and mRFP-positive dots.

### Lentivirus transduction

A lentivirus encoding prostaglandin-endoperoxide synthase 2 (PTGS2), also known as COX2, was purchased from GeneChem (Shanghai, China). Before transduction, equal numbers of HepG2 cells were plated in six-well plates. When the cells reached ~50% confluence, they were infected with lentivirus particles at an MOI of 10 for 16 h. To obtain stable clones, the infected cell lines were selected by puromycin (5 µg/ml) for 2 weeks. Once established, these stable clonal cells were subjected to either BBR treatment or mRFP-GFP-LC3 adenovirus transduction for further experiments.

### H&E staining and electron microscopy examination

Paraffin-embedded livers were cut into sections of 5 µm in thickness and stained with H&E as previously described^48^. A JEOL-1010 transmission electron microscope was used for examining the liver tissue samples as previously described^48^.

### Immunofluorescence staining

HepG2 cells were fixed with 4% paraformaldehyde and then blocked with a solution containing 5% bovine serum albumin and 0.02% Triton-X 100. After incubation overnight at 4 °C with primary antibodies against LC3B, p62, COX2, AKT, p-AKT, or LAMP-1, cells were washed with PBS and then further incubated for 90 min away from light with an appropriate fluorescence-conjugated secondary antibody. Nuclei was stained with DAPI (Invitrogen, Carlsbad, CA, USA). Finally, images of the cells were acquired by a fluorescence microscope (Axio Imager M2; Carl Zeiss Microscopy GmbH, Germany) or a confocal microscope (LSM-710; Carl Zeiss Microscopy GmbH).

### Filipin staining

Filipin III (2 mg) was dissolved in 800 µl of DMSO as a 50× stock solution. After treatment, HepG2 Cells were washed twice with cold PBS and fixed with 4% paraformaldehyde for 15 min. The samples were then stained away from light for 2 h with a 50 μg/ml filipin III working solution in PBS. Images were acquired using a Zeiss LSM-710 confocal microscope.

### Quantification of arachidonic acid metabolites by LC-MS

Liver tissue samples (50 mg each) from mice in the chow, AD, and AD + BBR diet groups were subjected to a determination of AA metabolites by LC-MS according to previously described procedures^49^.

### Western blot analysis

Mouse or human liver homogenates and HepG2 cell lysates were prepared, and their protein concentrations were determined with the BCA method. Protein samples (20 µg each) were loaded and separated on 10% SDS-PAGE. After the proteins were transferred onto polyvinylidene fluoride membranes (Millipore, Billerica, MA, USA), the membranes were blocked, incubated overnight at 4 °C with an appropriate primary antibody, and then incubated for 2 h at room temperature with an appropriate secondary antibody. Protein brands were visualized using ECL Plus reagent (Beyotime, Shanghai, China). Each experiment was replicated at least three times, and the acquired images were quantified by ImageJ software.

### RNA-seq

Total RNA was extracted from liver tissues using TRIzol reagent (Invitrogen). The corresponding cDNA libraries were constructed using the TruSeq Stranded Total RNA LT Sample Prep Kit (Illumina, Santiago, CA, USA) by Genergy Biological Technology Limited Co. (Shanghai, China) and sequenced with Illumina Hiseq3000. RNA-Seq reads were aligned to the reference data downloaded from UCSC (version hg19), and the RPKM method was utilized to normalize the reads that exclusively mapped to a gene, to quantify the transcript levels^50,51^.

### Quantitative real-time PCR

Total RNA from mouse livers were extracted by TRIzol reagent (Invitrogen), and then reverse-transcribed into cDNA using a High Capacity cDNA Reverse Transcription Kit (Applied Biosystems Foster City, CA, USA). Power Mix Sybr Green Master Mix (Applied Biosystems) was used for quantitative real-time PCR^52^ to determine the gene expressions of key enzymes involved in the AA metabolism (primer sequences are available upon request). The relative mRNA expression was then calculated with the ΔΔCt method using GAPDH as the internal control.

### Statistics

All the data are presented as the mean ± SEM. The comparisons among multiple groups were performed using a one-way ANOVA with a post-hoc test (Tukey’s multiple comparison test). A *p*-value of less than 0.05 was considered statistically significant. All the statistical tests were performed with IBM SPSS statistics 21.0 software.

## Electronic supplementary material

Supplementary Tables

Supplementary Figure 1

Supplementary Figure 2

Supplementary Figure 3

Supplementary figure legends
