# Supplementary material for: Berberine ameliorates blockade of autophagic flux in the liver by regulating cholesterol metabolism and inhibiting COX2-prostaglandin synthesis
Source: Cell Death Dis. 2018 Aug 1;9(8):824. doi: 10.1038/s41419-018-0890-5 (PMC6070517; doi:10.1038/s41419-018-0890-5)
Supplement: Supplementary file 1 — Supplementary Tables [file 41419_2018_890_MOESM1_ESM.docx]

**Supplementary Tables**

**Table 1. Demographic characteristics of the patients (means ±SEM)**

|  | Low cholesterol group | High cholesterol group |
| --- | --- | --- |
| Sex (Male/Female) | 3/3 | 3/3 |
| Age (years) | 48.8±5.8 | 47.3±4.3 |
| BMI (kg/m^2^) | 23.3±1.0 | 24.6±1.8 |
| Hepatic cholesterol  (μg/mg tissue) | 11.8±0.4 | 19.6±0.9** |

** High cholesterol group vs low cholesterol group: *P* < 0.01

**Table 2. Reagent information**

| Reagents | Manufacturer | Catalog Number |
| --- | --- | --- |
| Berberine chloride (BBR) | Sigma-Aldrich | PHR1502 |
| Chloroquine (CQ) | Sigma-Aldrich | C6628 |
| Wheat Germ Agglutinin (WGA), | Sigma-Aldrich | L4895 |
| Water-soluble Cholesterol | Sigma-Aldrich | C4951 |
| Prostaglandin E2 (PGE2) | Sigma-Aldrich | P0409 |
| Dimethyl Sulphoxide(DMSO) | Sigma-Aldrich | D2650 |
| Celecoxib | Sigma-Aldrich | 1098504 |
| Sodium Orthovanadate | APExBIO | A8524 |
| Filipin III | APExBIO | B6034 |

**Table 3. Antibody information**

| Antibodies | Manufacturer | Catalog Number |
| --- | --- | --- |
| LC3B | Proteintech | 18725-1-AP |
| LAMP-1 | Proteintech | 21997-1-AP |
| NPC2 | Proteintech | 19888-1-AP |
| SQSTM1(p62) | Proteintech | 66184-1-Ig |
| AKT | Cell Signaling Technology | 4804 |
| p-AKT(Ser473) | Cell Signaling Technology | 4060 |
| mTOR | Cell Signaling Technology | 2972 |
| p-mTOR(Ser2448) | Cell Signaling Technology | 5536 |
| Cox2 | Cell Signaling Technology | 12282 |
| GAPDH | Cell Signaling Technology | 5174 |
| CYP7A1 | Abcam | ab65596/ab78847 |
| SCP2 | Abcam | ab140126 |
| STARD4 | Abcam | ab202060 |
| Alexa Fluor 488 | Abcam | ab150077 |
| Alexa Fluor 594 | Abcam | ab150116 |
