# Supplementary figures and images for: Berberine ameliorates blockade of autophagic flux in the liver by regulating cholesterol metabolism and inhibiting COX2-prostaglandin synthesis
Source: Cell Death Dis. 2018 Aug 1;9(8):824. doi: 10.1038/s41419-018-0890-5 (PMC6070517; doi:10.1038/s41419-018-0890-5)

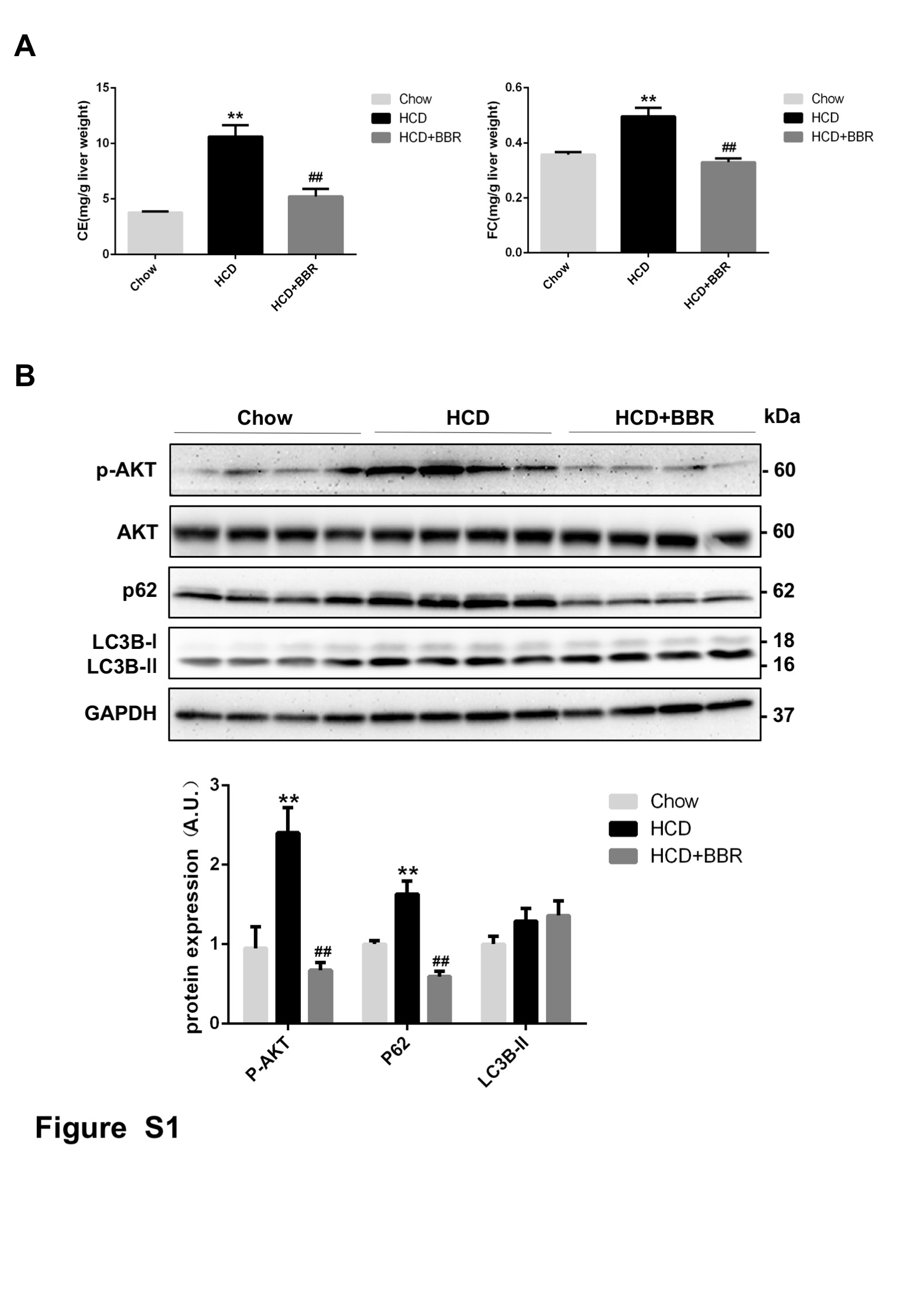

Supplement: Supplementary file 2 — Supplementary Figure 1 [file 41419_2018_890_MOESM2_ESM.jpg]

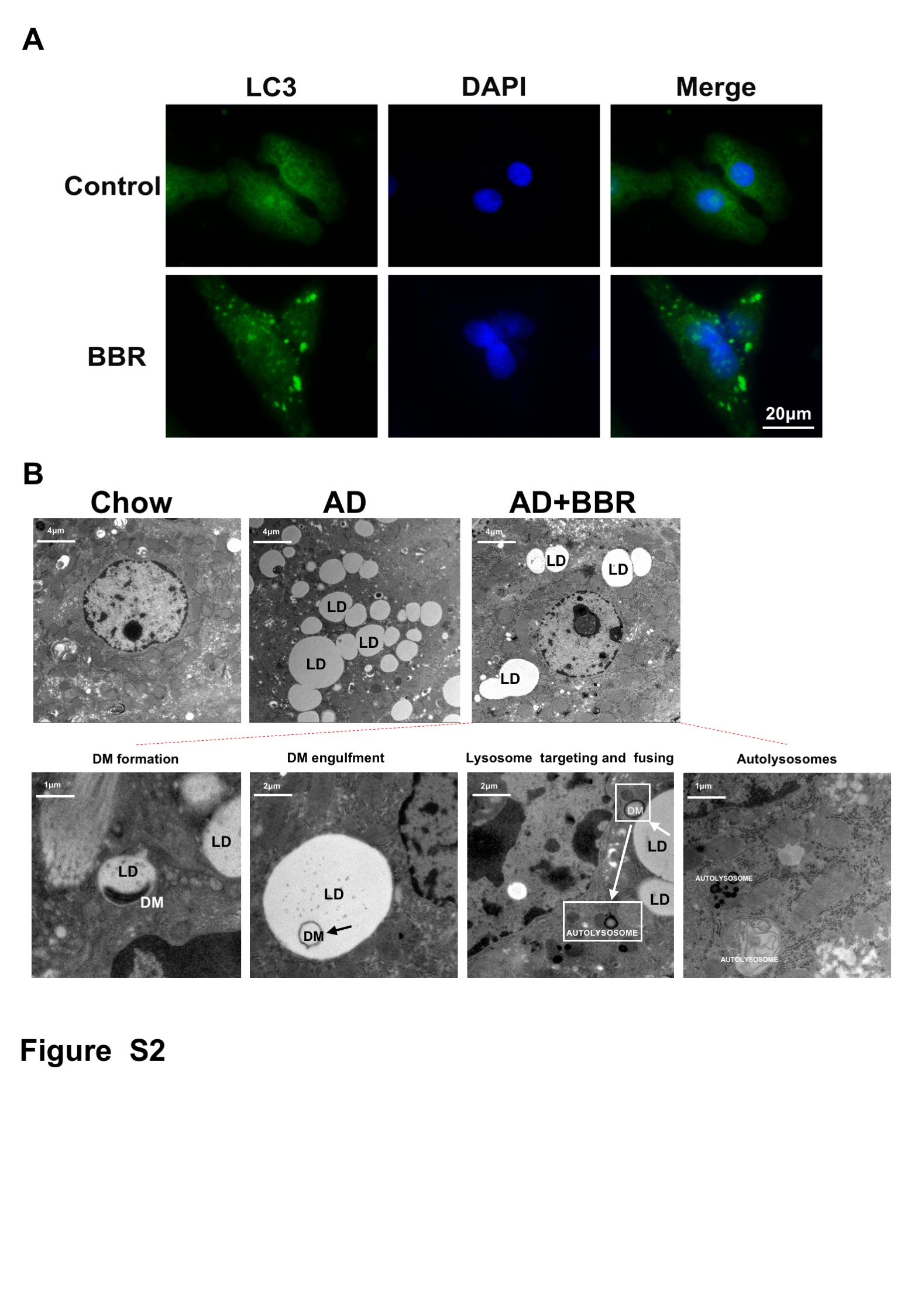

Supplement: Supplementary file 3 — Supplementary Figure 2 [file 41419_2018_890_MOESM3_ESM.jpg]

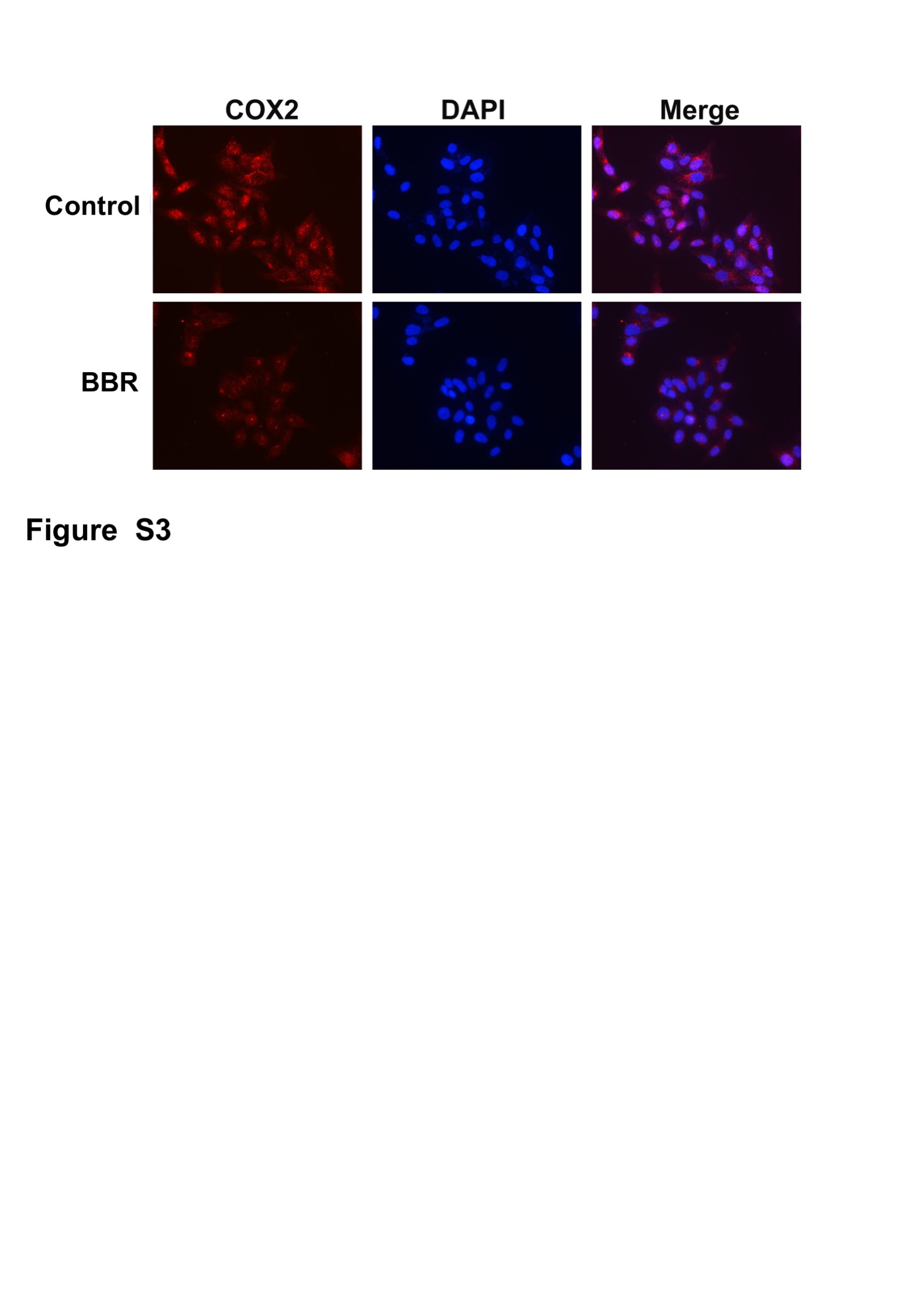

Supplement: Supplementary file 4 — Supplementary Figure 3 [file 41419_2018_890_MOESM4_ESM.jpg]
