# Supplementary material for: Berberine ameliorates blockade of autophagic flux in the liver by regulating cholesterol metabolism and inhibiting COX2-prostaglandin synthesis
Source: Cell Death Dis. 2018 Aug 1;9(8):824. doi: 10.1038/s41419-018-0890-5 (PMC6070517; doi:10.1038/s41419-018-0890-5)
Supplement: Supplementary file 5 — Supplementary figure legends [file 41419_2018_890_MOESM5_ESM.docx]

**Supplementary materials**

**Supplementary Figure Legends**

**Figure S1**

**A.** Cholesterol levels in the livers of mice fed with high cholesterol diet (HCD) alone or HCD with berberine (BBR). **B.** Expression of the p-AKT, p62, and LC3II proteins in the livers of mice fed with different diets. **: *p* < 0.01 compared with chow diet. ##: *p* < 0.01 compared with HCD.

**Figure S2**

**A**. Immunofluorescence staining of LC3B in HepG2 cells treated with berberine (BBR, 20 µg/ml for 8 h). **B**. Transmission electron microscopic appearance of liver tissue from mouse fed with chow, atherogenic diet (AD) or AD+BBR (upper) and typical double membrane structures (DM), DM engulfment in lipid droplets (LD), fusion with lysosomes, and autophagosome formation in mouse liver sections from mice in the AD + BBR group (lower).

**Figure S3**

Immunostaining of the COX2 protein in HepG2 cells treated with berberine (BBR, 20 µg/ml) for 8 h.
